# Upstream and Downstream of Recombinants Biomolecules to Health Care Industry

**DOI:** 10.1155/2016/9374847

**Published:** 2016-08-10

**Authors:** Priscila G. Mazzola, Arthur Cavaco-Paulo, Jorge G. Farías, Jorge F. B. Pereira

**Affiliations:** ^1^Faculty of Pharmaceutical Sciences, University of Campinas (UNICAMP), 13083-859 Campinas, SP, Brazil; ^2^Departamento de Engenharia Biológica, Universidade do Minho, Campus de Gualtar, 4710-057 Braga, Portugal; ^3^Facultad de Ingeniería y Ciencias, Departamento de Ingeniería Química, Universidad de la Frontera, Casilla 54-D, Temuco, Chile; ^4^School of Pharmaceutical Sciences, Universidade Estadual Paulista (UNESP), 14800-903 Araraquara, SP, Brazil

Biotechnology processes are the unique feasible way for the production of some pharmaceutical active principles. Thus, developments in molecular biology, recombinant techniques, separation, and purification methods have a primordial role because of the innovative characteristic and economic impact in obtaining these new drugs through biotechnological approaches. This special issue compiles a series of relevant studies on different biotechnological fields and applications, reporting up-to-date developments on downstream and upstream biopharmaceuticals.

Summarizing the results reported in the manuscripts published here, our readers may find further insights through a series of fields, from the most fundamental genetic approaches to the general aspects of biological and biochemical engineering. A complete study proposed by S. Zhang et al. applied next-generation RNA sequencing and developed a method to analyse the mutation rate of the mRNA of Chinese hamster ovary producing monoclonal antibodies, which are widely used for the production of biological therapeutics. Following the concept of monoclonal antibodies, E. Sasso et al. have presented a research study where they expanded the availability of monoclonal antibodies interfering with hepatitis C infection in hepatocytes. The results of these authors report an effective sequencing approach for library screening, demonstrating the successful conversion of recovered clones to active immunoglobulins. This novel approach allows rapid and cheap isolation of antibodies for virtually any native antigen involved in human diseases, for therapeutic and/or diagnostic applications.

On the other hand, to clone and express *γ*-polyglutamic acid (*γ*-PGA) synthetase gene in* B. subtilis*, B. Lin et al. have constructed a plasmid, which allowed the recombinant microorganism the synthesis of *γ*-PGA into the fermentation broth. This approach has potential industrial applications since *γ*-PGA is a new water-soluble biodegradable anionic polypeptide and, due to its interesting properties, such as nontoxicity, edibility, adhesiveness, film forming, and moisture retention capability, it can be a key compound for the health care industries. Also R. Niu and X. Chen reported a full-length cDNA, prokaryotic expression, and antimicrobial activity of cloned haemoglobin (Hb) from* Urechis unicinctus*, a marine spoon worm and economically important seafood. Their results elucidate the structure and potential function of Hb, which may help to understand the immune defense mechanism of invertebrates and to give some new insights into antimicrobial peptides for drug discovery and disease control in* U. unicinctus *aquaculture. Following the same concept, in “Enhanced and Secretory Expression of Human Granulocyte Colony Stimulating Factor by* Bacillus subtilis SCK6*,” S. Bashir et al. describe a simplified approach for enhanced expression and secretion of granulocyte colony stimulating factor (GCSF), a human cytokine, in the culture supernatant of* B. subtilis SCK6* cells. Their results have shown that after expression and purification the protein has a biological activity similar to the commercial preparation of GCSF. The last two works of this issue are aimed at the evaluation of stability of biomolecules and their accurate quantification, respectively. Formulating appropriate storage conditions for biopharmaceutical proteins is essential for ensuring their stability and thereby their purity, potency, and safety over their shelf life. With that in mind B. K. Chavez et al. employed a model murine IgG3 produced in a bioreactor and evaluated multiple formulation compositions. These studies have evaluated the antibody stability in a series of conditions using an experimental design approach, an optimized formulation being identified in which the stability was substantially improved under long-term storage conditions and after multiple freeze/thaw cycles. The last work is focused on the importance of proteases in the biotechnological and pharmaceutical industries, and, consequently, the determination of optimum conditions and the development of a standard protocol are critical during selection of a reliable method to determine its bioactivity. With that in mind, D. F. Coêlho et al. employed a quality control theory to validate a modified version of a method proposed in 1947, presenting a validated protocol that offers a significant improvement, given that subjective definitions are commonly used in the literature and this simple mathematical approach makes it clear and concise.

The quality of the results and protocols compiled in this issue have caught our interest, and we hope that these will help researchers and biotechnology-related professionals to develop more exciting science regarding the improvement of the human health and the sustainability and safety of the biotechnological industry.

*Priscila G. Mazzola* *Arthur Cavaco-Paulo* *Jorge G. Farías* *Jorge F. B. Pereira*

